# An Evaluation of Phenolic Compounds, Carotenoids, and Antioxidant Properties in Leaves of South African Cultivars, Peruvian 199062.1 and USA's Beauregard

**DOI:** 10.3389/fnut.2021.773550

**Published:** 2021-11-26

**Authors:** Charmaine J. Phahlane, Sunette M. Laurie, Tinotenda Shoko, Vimbainashe E. Manhivi, Dharini Sivakumar

**Affiliations:** ^1^Phytochemical Food Network Research Group, Department of Crop Sciences, Tshwane University of Technology, Pretoria, South Africa; ^2^Agricultural Research Council-Vegetable, Industrial and Medicinal Plants (ARC-VIMP), Pretoria, South Africa

**Keywords:** phytochemicals, leafy vegetable, caffeoylquinic acid, β-carotene, antioxidant activity

## Abstract

In this study, leaves of sweet potato cultivars from South Africa (“Ndou,” “Bophelo,” “Monate,” and “Blesbok”), “Beauregard,” a sweet potato cultivar from the USA, and a Peruvian cultivar “199062. 1” were analyzed using UPLC/QTOF/MS and chemometrics, with the aim of characterizing the locally developed sweet potato cultivars and comparing them with already well-known established varieties on the market. A set of 13 phenolic compounds was identified. A partial least squares discriminant analysis, a hierarchical cluster analysis, and variables importance in projection were used to successfully distinguish sweet potato varieties based on their distinct metabolites. Caffeic acid enabled to distinguish Cluster 1 leaves of varieties (“Beauregard” and “Ndou”) from Cluster 2 (“199062.1,” “Bophelo,” “Monate,” and “Blesbok”). The leaves of “Bophelo” contained the highest concentrations of rutin, quercetin 3-*O*-galactoside, 3-caffeoylquinic acid (3-CQA), (5-CQA), 1,3 dicaffeoylquinic acid (1,3-diCQA), 1,4-diCQA, and 3,5-diCQA. Furthermore, Bophelo leaves showed the highest antioxidant activities (FRAP 19.69 mM TEACg^−1^ and IC_50_ values of (3.51 and 3.43 mg ml^−1^) for DPPH and ABTS, respectively, compared to the other varieties. Leaves of “Blesbok” contained the highest levels of β-carotene (10.27 mg kg^−1^) and zeaxanthin (5.02 mg kg^−1^) on a dry weight basis compared to all other varieties. This study demonstrated that the leaves of local cultivars “Bophelo” and “Blesbok” have the potential to become functional ingredients for food processing.

## Introduction

Sweet potatoes (*Ipomoea batata* L. Lam.) are dicotyledonous plants of the Convolvulaceae family (1). Due to their high yield, drought resistance, and ability to grow in a cultivar of climates and conditions, leafy vegetables, such as sweet potato leaves, are becoming popular as a food security crop in developing countries (2). During the summer season, the leaves can be eaten as green leafy vegetables, possibly alleviating food shortages (2). The elements Na (8.06–832.31 mg 100 g^−1^ dry weight (DW), Mg (220.2–910.5 mg 100 g^−1^ DW), K (479.3–4,280.6 mg 100 g^−1^ DW), Ca (229.7–1,958.1 mg 100 g^−1^ DW, and P (131.1–2,639.8 mg 100 g^−1^ DW) are abundant in sweet potato leaves (3). The leaves, which flourish in poor, wet, and rich soil, can be cropped continuously until the root vegetables are harvested (3). While phytochemical content in sweet potato has been investigated, most of the research has concentrated on β-carotene with little information on variations in total phytochemicals and antioxidant activity among local cultivars.

Sweet potato leaves have been reported to contain phenolics in high levels, which make them superior to other commercial vegetables. Unlike spinach, cabbage, broccoli or kale, sweet potato leaves contain more polyphenols (4). Sweet potatoes high in polyphenol content are increasingly popular with health-conscious consumers (5). Sweet potato leaves, therefore, greatly contribute to the availability of food as well as bioactive compounds for consumers. As a functional food, sweet potato leaves contain a variety of bioactive compounds that have health-promoting properties. Polyphenols and carotenoids are among the compounds in sweet potato leaves reported to have beneficial effects on health (2). The functional compounds found in sweet potato leaves are responsible for a variety of biological functions (antioxidant, anticancer, antimutagenic, immune modulation, and liver protection) (2). As a functional ingredient, powdered sweet potato leaves can be used in food products, such as beverages in the food industry (6). In general, rural populations consume boiled or blanched sweet potato leaves. The sweet potato could become a profitable leafy vegetable crop if appropriate varieties were available or developed.

To date, the Agricultural Research Council of South Africa has released 25 cultivars due to its breeding program (7). “Blesbok,” “Bosbok,” and “Ribbok” are the main commercialized cultivars currently grown in South Africa, with a cream flesh in most cases (5). On the informal market, the most popular cultivars are “Ndou” (cream fleshed) and “Bophelo” (orange fleshed) (7), while the Peruvian cultivar 199062.1 and the “Beauregard” from the USA are being promoted. Specifically, the breeding effort aims to improve traits of interest to resource-poor farmers, such as high-dry matter content combined with high yield, as well as β-carotene content and resistance to drought and disease (7). However, very limited information is available on the content of phenolic compounds in locally used sweet potato cultivars. Thus far, a study by Nyati et al. (8) promoted the dual-purpose use of cultivar “Bophelo” in South Africa based on the iron, zinc, and β-carotene content. Therefore, for a full understanding of the composition of bioactive compounds and health benefits of the leaves for commercialization of locally grown sweet potato cultivars, it is important to know the predominant bioactive compounds.

In sweet potato leaves, caffeic acid and caffeoylquinic acid derivatives are the main phenolic acids (2). Specific genotypes and stages of leaf development influence these constituents (2). The amount of light exposure affects the concentration of phenolic components of sweet potato leaves (2). Compared with oven drying at 70 or 100°C, freeze-drying produced the most caffeoylquinic acid derivatives in sweet potato leaves (9). Moreover, the amount of lutein in sweet potato varieties varies from 34 to 68 mg 100 g^−1^, which makes it the main carotenoid component of sweet potato leaves (10). A strong antioxidant capacity is demonstrated by caffeoylquinic acid derivatives (11). However, it is essential to identify the specific caffeoylquinic acid present in the leaves of sweet potato cultivars that contribute to antioxidant activity by analyzing the correlation coefficients between two variables. As sweet potato leaves contain bioactive compounds, an assessment of the leaf compositions of the various cultivars grown locally in comparison to the cultivars already available on the market, such as “Beauragurad” and Peruvian “199062.1,” is necessary.

These three objectives serve as the basis for this study. The first objective of the study was to use metabolomic chemometrics to elucidate and characterize the leaves of locally developed sweet potato varieties and compare them with the cultivars “Beauregard” from the United States and the Peruvian cultivar “199062.1.” The second objective was to compare the antioxidant properties in the leaves of domestic cultivars, “Beauregard,” and Peruvian cultivar “199062.1.” The third objective was to compare carotenoid components in the leaves of local sweet potato cultivars with those in the Peruvian cultivar “199062.1” and the “Beauregard” cultivar.

## Materials and Methods

### Chemicals

The following chemicals used in this study: 2.2′-diphenyl-1-picrylhydrazyl (DPPH), methanol, trolox, potassium persulfate, sodium acetate trihydrate, persulfate sodium acetate, 2,4,6-tris(2-pyridyl)-1,3,5-triazine (TPTZ), FeCl_3_·6H_2_O, acetone, hexane, isopropyl alcohol, Folin-Ciocalteau reagent, Na_2_CO_3_, gallic acid, HCl, NaOH, diethyl ether, ethyl acetate, phosphate buffer, butylated hydroxytoluene (BHT), 2,2′-Azino-bis (3-ethylbenzothiazoline-6-sulfonic Acid) (ABTS), acetic acid, Na_2_SO_4_, acetonitrile, methanol, *N*-hexane formic acid, NH_4_OH, analytical standards (chlorogenic acid, neochlorogenic acid, caffeic acid, quercetin-3-*O*-glycoside, ferulic acid, vanillic acid, p-coumaric acid, β-carotene, lutein, gallic acid, zeaxanthin) were purchased from Sigma Aldrich, Johannesburg, South Africa.

### Plant Material

**Figure 1:**
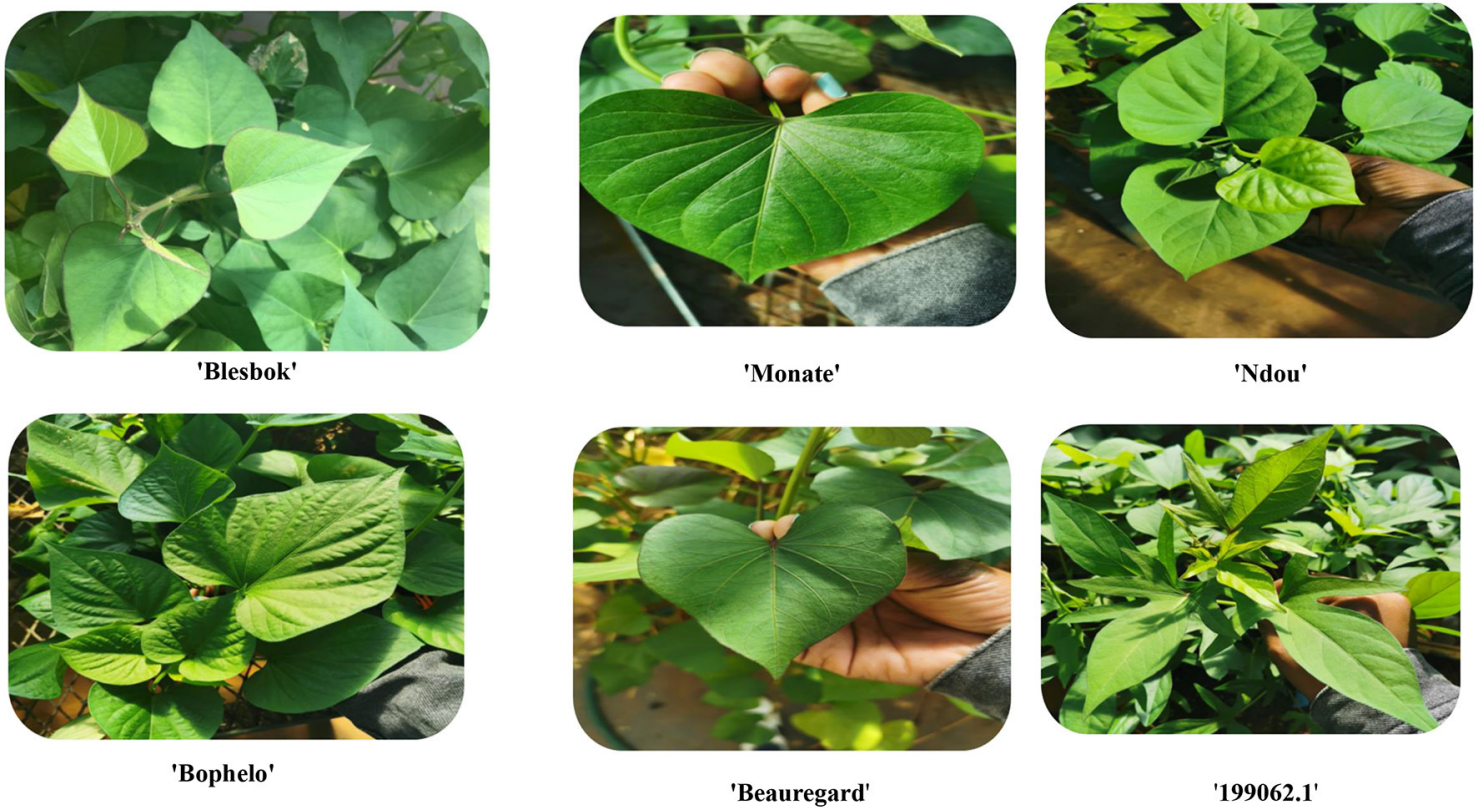
Leaves of different cultivars of sweet potatoes.

The leaves of four cultivars of sweet potato (*Ipomoea babatas* L.) developed in South Africa (orange-fleshed storage roots “Bophelo,” cream-fleshed “Monate,” “Ndou,” “Blesbok”) and the USA's “Beauregard” cultivar (orange fleshed) and Peru's “1999062.1” cultivar (yellow orange flesh) were obtained from Agricultural Research Council (ARC-VIMP), Roodeplaat, Pretoria (Figure 1). The planting of the cultivars, according to standard production practices, took place in the middle of October 2020, with an average temperature of 25–31°C. The random harvesting of the leaves, up to the fifth leaf from the tip of the vines, occurred early morning. Leaves free from damage and decay were sorted and the dirt removed by washing under running tap water. Afterwards, the leaves were freeze-dried (United Scientific freeze dryer, Model FM25XL-70 at −55°C), grounded into a fine powder and stored at −20°C for biochemical analysis.

### Total Phenolic Content (TPC)

Total phenolic content was measured by the Folin-Ciocalteau method (12). A freeze-dried sample (1 g) was extracted with 10 ml of 80% methanol by shaking in a magnetic stirrer for 2 h. Afterward, an aliquot of 100 μl of the extract was mixed with 200 μl of 10% Folin-Ciocalteau, and then 800 μl of 7.5% Na_2_CO_3_ was added to the mixture, and the absorbance was measured at 736 nm using a spectrophotometer. Quantification was done using gallic acid as a reference standard prepared at concentrations ranging from 0 to 100 μg ml^−1^. Total phenolic content was expressed as milligrams gallic equivalent per kilogram (mg GAEkg^−1^) on a dry weight basis (DW).

### Total Antioxidants Activities

The Ferric Reducing Antioxidant Power (FRAP) assay was performed according to the procedure described by Seke et al. (13) using the FRAP reagent, which was made up of 10 mmol·L^−1^ solution of TPTZ in 40 mM of HCl, 20-mM solution of FeCl_3_·6H_2_O and a 20-mM acetate buffer (pH 3.6) mixed in a 1:1:10 ratio, respectively. The sample (0.1 g) was homogenized with 80% methanol and 20 μl of the sample and 150 μl of FRAP reagent and incubated for 10 min, and, thereafter, the absorbance was measured at 593 nm on a microplate reader. Trolox solution ranging from (0 to 30 mM) was prepared for quantification as a reference standard; results were expressed as mM TEACg^−1^.

The radical scavenging activity was assessed using the 2,2′-diphenyl-1-picrylhydrazyl radical (DPPH) scavenging ability assay with slight modifications, according to the method described by Seke et al. (13), using.1 g of a freeze-dried sample mixed with 1 ml of 80% methanol. The sample mixture was centrifuged at 3,000 × *g* for 5 min at 4°C using a Hermle centrifuge (Model Hermle Z326k, HermleLabortechnik GmbH, Wehingen, Germany). Different sample concentrations (100 μl) were made by serial dilution (0–10 mg ml^−1^) and 200 μl DPPH solution (13 μl DPPH ml^−1^ methanol) were added to each well, and the absorbance was measured at 517 nm after incubating for 20 min at ± 25°C. The % inhibition was calculated using the equation.

$$\begin{array}{l} \text{DPPH \% inhibition }=\;\left(\text{A}0-\text{A}1/\text{A}0\right)\;\times \;100 \end{array}$$

Where A0 is the absorbance of the DPPH radical solution, and A1 is the absorbance of a sample. The IC_50_ (mg ml^−1^) was calculated from the graph of the inhibition percentage vs. the concentration.

2,2′-Azino-bis (3-Ethylbenzothiazoline-6-Sulfonic Acid) (ABTS^+^) radical scavenging was performed based on the method previously described by Seke et al. (13). The production of the ABTS radical cation (ABTS^+^) was determined by the reaction of the ABTS stock solution (7 mM) with 4.9-mM potassium persulphate at the ratio of 1:1 and allowing the mixture to incubate in the dark at 25°C for 12-16 h before use. An aliquot of 40 μl of a sample (different concentrations from 0-10 mg ml^−1^ made by serial dilution) was pipetted into 200 μl of the ABTS^+^. The mixture was incubated in the dark in a 96-well microplate reader at 37°C for 10 min; the decrease in absorbance at 734 nm was measured. The % inhibition was calculated using the equation.

$$\begin{array}{l} \text{ABTS \% inhibition }=\;\left(\text{A}0-\text{A}1/\text{A}0\right)\;\times \;100 \end{array}$$

Where A0 is the absorbance of the ABTS radical solution, and A1 is the absorbance of a sample. The IC_50_ (mg ml^−1^) was calculated from the graph of the inhibition percentage vs. the concentration.

### Untargeted Metabolites

The method of Mashitoa et al. (14) was used to extract and analyze metabolites without any changes. Briefly, freeze-dried samples of leaves (2 g) were homogenized with 15 ml of 80:20 methanol/water (v/v) at 25°C. The samples were vortexed for 1 min and then extracted by sonication (MRC ultrasonic cleaner) for 1 h, centrifuged at 2,000 × *g* using a Hermle centrifuge (Model Hermle Z326k, HermleLabortechnik GmbH, Wehingen, Germany), and the supernatant was taken for analysis. Analysis was done using a Waters Acquity Ultra performance liquid chromatograph (UPLC) hyphenated to a Waters Synapt G2 Quadrupole time of flight (QTOF) mass spectrometer (MS) (Waters, Milford, MA, USA). Concentrations of phenolic compounds were determined using reference standards catechin, epicatechin, caffeic acid, and chlorogenic acid and rutin to quantify compounds based on the areas of their extracted mass chromatograms. Table 1 presents the chemical formulas, mass fragments, and UV absorbance of the tentatively identified phenolic compounds.

**Table 1:** Tentative identification of phenolic compounds in the leaves of different sweet potato cultivars by UPLC–QTOF/MS.

**Peak**	**Retention time (min)**	**[M-H]-**	**M-H formula**	**Error (ppm)**	**MSE fragments**	**UV**	**Tentative identification**
1	4.068	353.08893	C_16_H_18_O_9_	−3.17	191, 179, 173, 135	324	Neochlorogenic acid (5-CQA)
2	4.525	369.08224	C_16_H_18_O_10_	1.31	207, 192, 167	324	5-Hydroxy-6-methoxycoumarin 7-glucoside
3	5.123	595.16675	C_27_H_32_O_15_	0.08	385, 355, 285	290, 330	Eriodictyol 7-O-neohesperidoside (Neoeriocitrin)
4	5.235	179.03531	C_9_H_8_O_4_	−1.82	179, 135	290, 323	Caffeic acid
5	5.393	353.08524	C_16_H_18_O_9_	7.28	191, 179, 173, 161, 135	318	Chlorogenic acid (3-CQA)
6	5.967	625.14038	C_27_H_30_O_17_	1.04	300, 191, 179, 135	339	Quercetin 3-glucosyl-(1->2)-galactoside
7	6.279	380.99002	C_18_H_6_O_10_	−3.41	301, 179, 151	335	Quercetin derivates
8	6.655	609.14282	C_27_H_30_O_16_	5.41	300, 151	255, 353	Quercetin-3-O-rutinoside (Rutin)
9	6.896	463.08786	C_21_H_20_O_12_	0.74	300, 271, 285, 179, 151	255, 355	Quercetin 3-galactoside (Q-3-GA)
10	7.116	515.11896	C_25_H_24_O_12_	1.06	353, 191, 179, 135	324	3,5-Dicaffeoyquinic acid (3,5-diCQA)
11	7.344	515.11835	C_25_H_24_O_12_	2.24	353, 300, 173, 135	324	1,3-Dicaffeoyquinic acid (1,3-diCQA)
12	7.647	515.12085	C_25_H_24_O_12_	−2.61	353, 300, 191, 173, 135	324	1,4-Dicaffeoyquinic acid (1,4-diCQA)
13	8.336	515.11902	C_25_H_24_O_12_	0.94	353, 300, 203, 191, 173, 179	324	4,5-Dicaffeoyquinic acid (4,5-diCQA)

### Extraction of Carotenoids

Carotenoids were extracted according to the method of Panfili et al. (15) with minor changes. Freeze-dried leaves (1 g) were extracted using 5 ml of acetone: hexane (1:1) with 0.1% butylated hydroxytoluene (BHT) by keeping them in a dark overnight in tightly closed tubes. The mixture was separated using a centrifuge (HermleLabortechnik, Germany Type 2326K, 2010) at 2,000 × g for 15 min at 25°C using a Hermle centrifuge (Model Hermle Z326k, HermleLabortechnik GmbH, Wehingen, Germany). Afterwards, the residue was rinsed with three additional 5-ml volumes of the extraction solvent, and centrifugation was done as before. The supernatants were pooled, dried with anhydrous sodium sulfate, filtered with a Whatman filter paper (No. 1) and evaporated to dryness under a stream of nitrogen. The extract was redissolved in 1 ml of isopropyl alcohol (10%) in n hexane. Quantification was done by using reference standards of each of β-carotene, zeaxanthin, and lutein at concentrations ranging from (0 to 100 μg ml^−1^) to quantify compounds based on their areas.

### Statistical Analysis

This study was laid out in a completely randomized design with 10 replicates per cultivar, and the experiments were repeated two times by harvesting the leaves in 2020 in December-January. The Genstat (VSN International, Hemel Hempstead, UK) for Windows 13th Edition (2010 version) analyzed the differences between the leaves of different sweet potato cultivars using a one-way ANOVA. To compare the means of the different biochemical components analyzed from the leaves of different sweet potato cultivars, the least significant difference test (LSD) was used, with *p* < 0.05. Each of the six sweet potato cultivars was replicated three times. The results are expressed as mean ± standard deviation. Data sets obtained from the UPLC-Q-TOF/MS analysis for three replicate samples of the leaves of different sweet potato cultivars were imported into MetaAnalyst 5.0 to perform partial least squares discriminant analysis (PLS-DA), variables importance in projection (VIP) scores, and heat maps. Pearson's correlation was conducted, and we used Pearson's correlation coefficients as a distance measure in the graph.

## Results and Discussion

### Total Phenols

**Figure 2:**
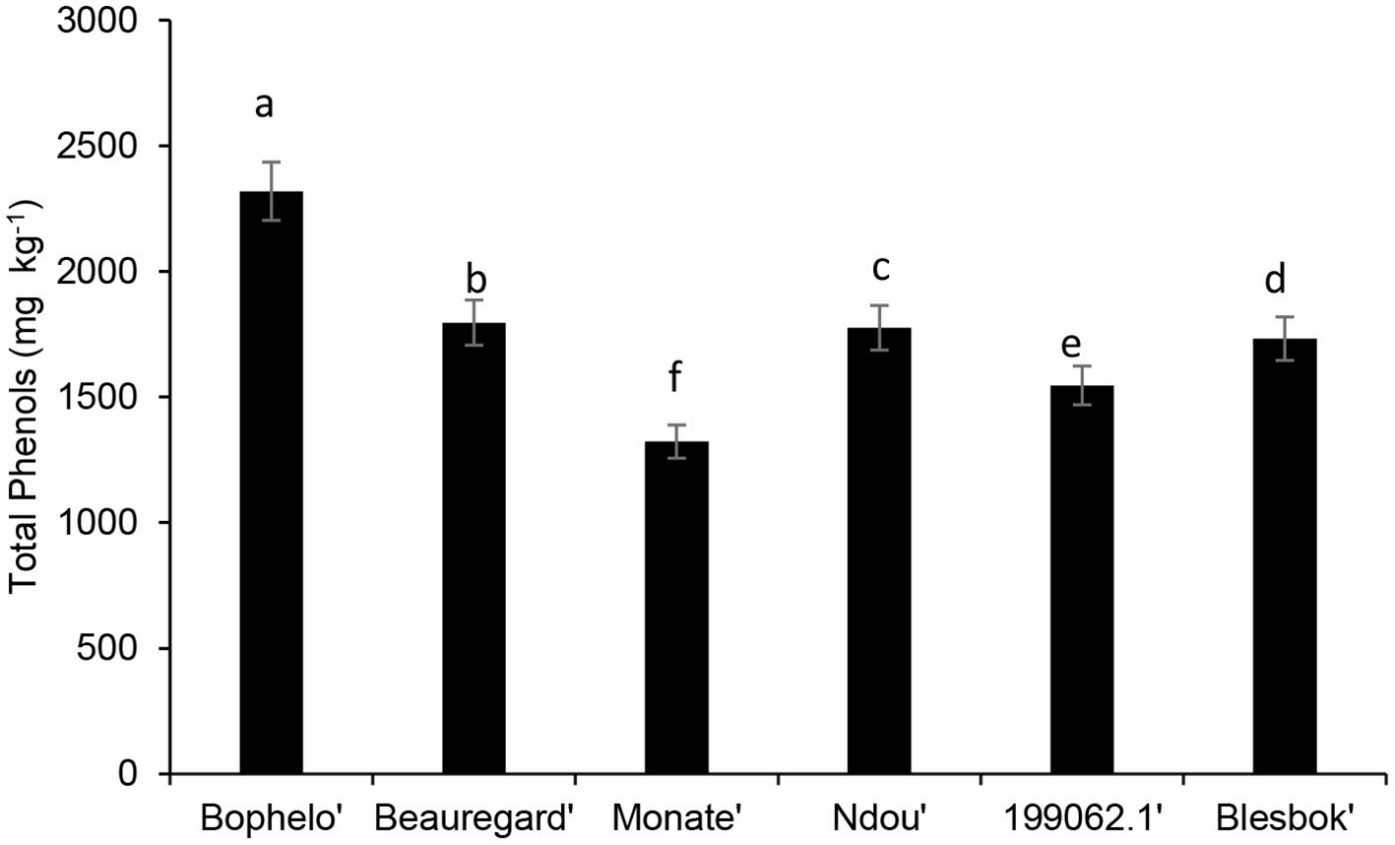
Comparison of the total phenolic content in the leaves of different South African sweet potato cultivar with the USA variety Beauregard and the Peruvian cultivar 199062.1. Bars with similar alphabetic letter are not significantly different at *p* < 0.05 according to Fisher's LSD test.

Figure 2 presents the results of the quantification of total phenolic content. Based on total phenolic content in the leaves of six sweet potato varieties, “Bophelo was the highest, followed by “Beauregard,” “Ndou,” “Blesbok,” “199062.1,” and, finally, “Monate.” The total phenol content of freeze-dried leaves of six sweet potato cultivars ranged from 2319.10 to 1322.76 mg kg^−1^. By contrast, Malaysian sweet potato leaves varied between 3,470 and 5,350 mg kg^−1^ in dry weight (11). Furthermore, eight sweet potato cultivars from Japan had leaf total phenolic compounds ranging from 6.3 to 13.5 g GAE100 g^−1^ dry weight, higher than the concretions found in the four South African cultivars and the “Beauregard” and 2000621. Jiang and Koh (16) reported that the leaves of six major North Korean sweet potato cultivars genetically engineered in South Korea contained an average of 650–1,910 g of phenols in 100 g of fresh weight; however, this has no comparison with our data generated on a dry weight basis. Reports of Islam et al. (17) showed that the highest phenolic content in the leaves of “1,389” sweet potato cultivars, lines, and genotypes preserved in the gene bank of NARCKO (National Agricultural Research Center of Kyushu Okinawa Region, Japan) was 6,190 mg kg^−1^ Islam et al. (17). Overall, the total phenolic content of all six sweet potato cultivars was higher than those reported for the common indigenous leafy vegetables *Amaranthus dubius* (516 mg kg^−1^), *Cleome gynandra* (268 mg kg^−1^), and *Cucurbita maxima* (394 mg kg^−1^) (18).

### An Untargeted Phenolic Metabolite Profile

Table 1 revealed 13 phenolic compounds tentatively identified by UPLC–QTOF/MS from the leaves of six sweet potato cultivars. The UPLC–QTOF/MS helped to identify caffeoylquinic acid derivatives [(neochlorogenic acid(5-CQA); chlorogenic acid 3-CQA, 3,5-dicaffeoylquinic acid (3,5-diCQA), 1,3-dicaffeoylquinic acid (1,3-diCQA), 1,4-dicaffeoylquinic acid (1,4-diCQA), and 4,5-dicaffeoylquinic acid (4,5-diCQA)], neoeriocitrin (eriodictyol 7-O neohesperidoside), caffeic acid, quercetin 3-glucosyl-(1->2)-galactoside, quercetin derivates, quercetin 3-*O*-galactoside (Q-3-GA), and quercetin-3-*O*-rutinoside (rutin) in the leaves of all six sweet potato varieties. Conversely, the leaves of nine sweet potato cultivars grown in the moderate climate of Poland contained seven polyphenolic compounds, including five caffeoyquinic acid derivatives-−5-CQA, 3-CQA, 4-cryptochlorogenic acid (4-CQA), 3,4-diCQA, 3,5-diCQA—and flavonoids, Q-3-GA and quercetin-3-*O*-glucoside (Q-3-GL) (19). The leaves of six major North Korean sweet potato cultivars genetically engineered in South Korea contained four phenolic compounds, 3CQA, (4,5-diCQA, 3,5-3,5-diCQA, and 3,4-diCQA (16). The observed differences in phenolic components are likely due to the genetic composition of the varieties examined, the climate conditions, and the maturity of the leaves harvested in this study (19). The Supplementary Figures 1A–N shows the UV spectrum MS, MS/MS spectrum, and the chemical structures of the tentatively identified compounds.

### Metabolomic and Chemometric Profiles

**Figure 3:**
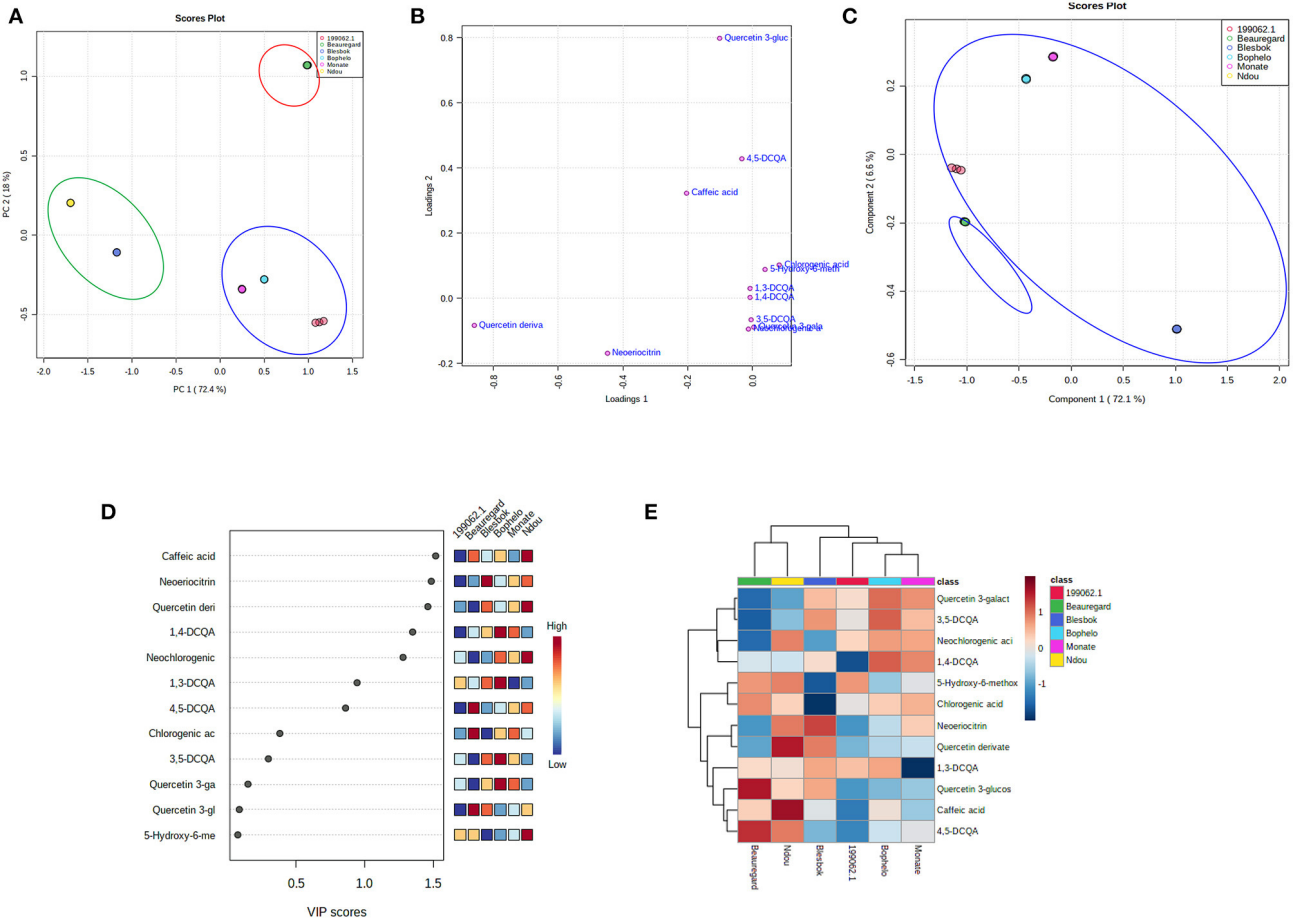
Statistical analyses of bioactive metabolites by Metaboanalyst 5.0 software. **(A)** An unsupervised PCA score plot of phenolic metabolites generated by UPLC-QTOF/MS analysis showing the separation of three clusters. F/MS analysis showing the separation of three clusters. **(B)** A PLS-DA score plot showing six sweet potato cultivars clustered into three groups. **(C)** PLS-DA score plots loaded with different phenolic compounds detected by UPLC-QTOF-MS. **(D)** In PLS-DA, metabolites are assigned VIP scores. The score they receive from low to high determines the importance of variables. The colored boxes on the right show the relative concentration of each of the metabolites. High red levels indicate high levels, and low blue levels indicate low levels. **(E)** Heat map. On the map, the colored areas correspond to the concentrations of different phenolic compounds found in different sweet potato cultivars. Each row represents a phenolic compound, and each column represents the leaf of sweet potato cultivar. Red indicates high levels, and blue indicates low levels.

Using the UPLC-Q-TOF/MS data, PCA analysis of unsupervised results helped to identify which sweet potato cultivars contain the most and fewest functional compounds (Figure 3A). Two-dimensional scatter plots between PC1 and PC2 explained 90.4% of the total variance (72.4 and 18%, respectively). In Figure 3A, three primary groups or clusters of sweet potato cultivars are distinguished based on their leaf phenolic compounds in a systematic and obvious way. As a result, these results confirm that the concentration of different phenolic compounds plays a key role in determining the classification of sweet potato leaves. The loading plot in Figure 3B indicates that the greater the distance between a point and its original point, the greater the contribution of the compound to the total variation. The compounds 5CQA, 1,3-diCQA, 1,3-,1,4-diCQA, 3,5-diCQA, Q-3-GA, and 4,5-diCQA were loaded positively on PC1 and separated the leaves of cultivars “199062.1,” “Bophelo,” and “Monate” from the rest. Quercetin derivatives and eriodictyol-7-O-neohesperidoside were loaded negatively on PC1, helping to separate the leaves of varieties “Blesbok” and “Beauregard” from the rest. Quercetin 3-glucosyl-(1->2)-galactoside accumulated on PC2 and separated the leaves of “Beauregard” from the others. Therefore, these nine compounds account for most of the variations found between the leaves of six sweet potato cultivars. Despite this, further information must be extracted from the data to provide more specific, meaningful information. Due to this, the complete data set was subjected to partial least squares discriminant analysis (PLS-DA) to determine changes in metabolites according to the type of sweet potato cultivar. PLS-DA has the advantage of not relying on a particular distribution, thus producing more accurate predictions and descriptive models (15). A good fit is observed for the PLS-DA model (*R*^2^ = 0.90), and its predictability is high (*Q*^2^ = 0.85), allowing us to forecast metabolite changes from the data. Principal component 1 (PC1) accounts for 72.1% of the total variation, while principal component 2 (PC2) accounts for 6.6% of the total variation both together contributing toward 78.1% (Figure 3C). PLS-DA was applied to classify the leaves of sweet potato cultivars by their 13 phenolic compounds. PLS-DA plots showed two large groups due to phenolic metabolite distribution. There are four types of sweet potato leaves in Cluster 1: “199062.1,” “Bophelo,” “Monate,” and “Blesbok.” “Leaves of “Beauregard” and “Ndou” were placed in Cluster 2. Based on the concept of proximity, it is clear that “199062.1,” “Bophelo,” “Monate,” and “Blesbok” share similar metabolites at higher concentrations. The entire dataset of identified metabolites was analyzed using hierarchical cluster analysis, and clusters of samples with similar chemical composition were shown, providing further evidence about the related metabolites associated with the leaves of different sweet potato cultivars. This analysis was accompanied by a heat map structure based on metabolite concentrations in all samples. The cladogram at the top of the heat map in Figure 3D confirms that there are two major clusters in the PLS-DA plot. Each cluster gram represented a row of data across each column of phenolic compounds as a color block, with dark red boxes representing higher levels of metabolites and dark blue boxes suggesting lower levels. Figure 3D shows the 13 metabolites identified in these two groups. The levels of quercetin 3-glucosyl-(1->2)-galactoside and 4,5-diCQA were higher in the leaves of “Beauregard.” Leaves of “Ndou” contained higher levels of caffeic acid and quercetin derivates. Leaves of “Blesbok” contained higher concentration of eriodictyol-7-O-neohesperidoside. Furthermore, the heat map represented the tendency of phenolic metabolite compositions in leaves of six sweet potato cultivars.

Additionally, we evaluated the contributions of each metabolite to the separation of groups using Variable Importance in Projection (VIP) scores. A VIP score is determined by summing the squares of the PLS loadings, which indicate how much Y-variance is explained across all dimensions and by adding the weighted sum of the PLS regression coefficients (20). To provide the most meaningful interpretation of the results, only the top metabolites with the highest VIP scores were considered (20) (Figure 3E). Among the top six metabolites with VIP scores >1 are caffeic acid, eriodictyol-7-O-neohesperidoside (Neoeriocitrin), quercetin derivates, 1,4-diCQA, quercetin derivates, eriodictyol-7-O-neohesperidoside 3,5-diCQA, and neochlorogenic acid (cis-3-CQA) (Figure 3E). Caffeic acid enabled us to distinguish Group 1 (leaves of cultivars “199062.1,” “Bophelo,” “Monate,” and “Blesbok”) from Group 2 (“Beauregard” and “Ndou”).

### Quantified Concentrations of Phenolic Compounds

The concentrations of 13 phenolic compounds determined from six cultivars of sweet potatoes are presented in Table 2. Among the leaves of six sweet potato cultivar, “Bophelo” contained the highest concentration of rutin, Q-3-GA, 3-CQA, 5-CQA, 1,3-diCQA, 1,4-diCQA, and 3,5-diCQA. Comparison sweet potato cultivar “Beauregard” showed higher concentrations of quercetin 3-glucosyl-(1->2)-galactoside, 3-CQA, and 4,5-diCQA in the leaves. However, Peruvian cultivar “199062.1” showed the highest concentration of 5-hydroxy-6-methoxycoumarin 7-glucoside and 1,3-diCQA. According to Jung et al. (1), the compound most abundant in sweet potato leaves is 5-neochlorogenic acid (5-CQA), and its amount depends on different cultivars. In contrast, Krochmal-Marczak (19) noted that the chlorogenic acid (3-CQA) was the dominant CQA derivative. Our study found 1,4-diCQA, 3,5-diCQA, and 3-CQA to be the dominant CQA derivatives in the leaves of all six sweet potato cultivars. The phenolic compounds in sweet potatoes have shown the ability to promote human health and can be used as functional foods (1). Therefore, the leaves of the local cultivar “Bophelo” have potential as a functional food. Reportedly, caffeoylquinic acid was antimutagenic in the *Salmonella ames* experiment when caffeoyl groups were bound to quinic acid (21). Additionally, out of the six CQA components, five components were found in higher concentrations in leaves of the local cultivar “Bophelo” while two caffeoylquinic acids (3-CQA, 4,5-diCQA) and one dicaffeoylquinic acid (1,3-diCQA) were detected in the highest concentrations in “Beauregard” and “199062.1” leaves, respectively. The dominant caffeoylquinic acids in the leaves of the local cultivar “Bophelo” were 1,4-DCQA and 3,5-DCQA; additionally, 3,5-DCQA was predominant in the leaves of the USA cultivars “Covington” and “Hernandez” (22). The compound 4-CQA was, however, not detected in the leaves of these sweet potato cultivars. It is likely that 4-CQA is affected by the maturity stage at harvest and is prominent at early maturity stages (19). Furthermore, caffeoylquinic acids and dicaffeoylquinic acids exhibit various biological activities in animals and plants (23).

**Table 2:** Comparison of different phenolic compounds in the leaves of four Southern African sweet potato (*Ipomoea batatas* L.) cultivars with “Beauregard” from the USA and Peruvian “199062.1.”

	**Orange fleshed storage roots**			**Cream fleshed roots**		
Phenolic components (mg/kg)	“Bophelo” leaves	“Beauregard” leaves	“199062.1” leaves	“Monate” leaves	“Ndou” leaves	“Blesbok” leaves
5-Hydroxy-6-methoxycoumarin 7-glucoside	6.96 ± 0.20c	9.59 ± 0.08a	9.59 ± 0.22a	7.92 ± 0.37b	9.86 ± 0.21a	5.33 ± 0.11d
Eriodictyol-7-O-Neohesperidoside (Neoeriocitrin)	15.94 ± 0.19d	9.50 ± 2.49e	9.35 ± 1.95e	24.43 ± 1.8c	33.86 ± 0.70b	40.26 ± 0.41a
Caffeic acid	41.89 ± 0.17b	44.82 ± 0.56b	26.76 ± 0.34e	34.10 ± 0.64d	65.07 ± 2.00a	39.81 ± 1.55c
Quercetin 3-glucosyl-(1->2)-galactoside	6.97 ± 0.14d	29.52 ± 0.18a	5.46 ± 0.69d	7.87 ± 1.26d	14.38 ± 0.69c	17.89 ± 0.54b
Quercetin derivates	1.56 ± 0.06c	0.26 ± 0.09c	0.60 ± 0.49c	2.24 ± 0.80c	31.13 ± 1.34a	15.98 ± 0.41b
Quercetin-3-O-rutinoside (Rutin)	32.27 ± 0.11a	5.63 ± 1.18d		13.59 ± 4.47c	5.51 ± 0.42d	
Quercetin 3-galactoside	25.04 ± 0.43a	21.33 ± 0.37e	23.73 ± 0.68c	24.68 ± 0.11a	22.09 ± 0.11d	24.19 ± 0.64b
Caffeoylquinic acid components						
Chlorogenic acid (3-CQA)	49.81 ± 0.70a	53.76 ± 0.73a	46.95 ± 0.97b	51.66 ± 0.41a	49.49 ± 1.59a	33.74 ± 0.16c
Trans-5-O-caffeoylquinic acid (Neochlorogenic acid) (cis-3-CQA)	80.49 ± 0.51a	70.11 ± 0.22e	78.04 ± 0.49c	79.89 ± 0.30b	81.02 ± 1.01a	72.23 ± 0.90d
1,3-Dicaffeoylquinic acid (1,3-diCQA)	29.76 ± 0.14a	28.23 ± 0.21b	29.04 ± 0.22a	21.56 ± 7.20c	27.93 ± 0.21b	29.71 ± 0.54a
1,4-Dicaffeoylquinic acid (1,4-diCQA)	49.69 ± 0.70a	47.47 ± 0.16b	44.92 ± 0.74c	49.20 ± 0.58a	47.38 ± 0.22b	48.05 ± 0.47b
3,5-Dicaffeoylquinic acid (3,5-diCQA)	47.90 ± 0.24a	43.06 ± 0.62e	45.86 ± 0.39c	46.71 ± 0.38b	44.60 ± 0.10d	47.10 ± 0.79b
4,5-Dicaffeoylquinic acid (4,5-diCQA)	17.62 ± 0.57c	29.09 ± 0.50a	12.62 ± 0.06d	18.66 ± 0.72c	25.35 ± 0.64b	14.77 ± 0.09d

*Means followed by the same letter within the row are not significantly different (p < 0.05), each of the samples was replicated three times, and the results are expressed as mean ± standard deviation*.

### Antioxidant Capacities

Table 3 compares the antioxidant activities of the leaf extracts of six South African sweet potato cultivars with the USA's Beauregard and Peru's “199062.1” varieties. FRAP, DPPH, and ABTS activities were the highest in leaves of the domestic sweet potato cultivar “Bophelo,” compared to the other domestic cultivars and the Peruvian cultivar “199062.1.” “Bophelo” leaves showed greater FRAP and DPPH scavenging activities than “Beauregard” leaves despite similar ABTS activity. According to Ghasemzadeh et al. (24), leaf extracts of sweet potato leaves grown in Malaysia with higher total phenolic compounds showed stronger radical scavenging. Compounds exhibiting strong antioxidant activity contain phenolic groups or a large number of conjugated hydroxyl moieties, which are capable of donating electrons to oxidizing radical species (20). It has been established that antioxidant molecules prevent cellular damage and macromolecular degradation by blocking the oxidation of other molecules (20).

**Table 3:** Comparison of the antioxidant activities of the leaf extracts of four South African sweet potato varieties with the USA's Beauregard and Peru's 199062.1.

	**Orange fleshed storage roots**			**Cream white fleshed roots**		
Antioxidant activity	“Bophelo” leaves	“Beauregard” leaves	“199062.1” leaves	“Monate” leaves	“Ndou” Leaves	“Blesbok” leaves
FRAP (mM TEAC/g)	19.69 ± 0.78a	18.71 ± 0.03b	17.65 ± 0.04c	17.56 ± 0.01c	17.83 ± 0.05c	17.81 ± 0.08c
DPPH (IC_50_ mg/ml)	3.51 ± 0.01d	4.22 ± 0.01c	4.99 ± 0.00ab	5.21 ± 0.00a	4.72 ± 0.03b	4.93 ± 0.07b
ABTS (IC_50_ mg/ml)	3.43 ± 0.00c	3.54 ± 0.02c	4.28 ± 0.04b	4.60 ± 0.01a	3.68 ± 0.05c	4.17 ± 0.03b

*Means followed by the same letter within the row are not significantly different at p < 0.05. Each of the sweet potato samples was replicated three times, and the results are expressed as mean ± standard deviation*.

A correlation analysis assesses the relationship between two variables by using statistical techniques. A high correlation coefficient indicates a strong relationship between two or more variables, while a low correlation indicates a weak relationship. In order to consider a correlation to be strong, we set a threshold of 0.5. The total phenol content and FRAP activity were strongly and positively correlated (*r* = 0.85, *p* < 0.05) in this study. ABTS radical scavenging activity (*r* = 0.75, *p* < 0.05) and DPPH scavenging activity (*r* = 0.88, *p* < 0.05) are also strongly correlated with total phenol content. Sweet potato leaves contain positive correlations between total polyphenol content and all caffeoylquinic acid derivatives, except for five caffeoylquinic acids and caffeic acids (3). Islam et al. concluded that the correlation between total phenolics and derivatives of the CQA may contribute to the improvement of desirable parameters in the selection of cultivars. Furthermore, a positive correlation (*r* = 0.69 *p* < 0.05) was found between 1,3-diCQA and total phenols in our study. A previous study by Danino et al. (23) demonstrated that 1,3-diCQA exhibits antioxidant properties. In their study, Danino et al. (23) showed that 1,3-diCQA has an IC_50_ of around 2-fold less than trolox, demonstrating greater antioxidant activity than trolox. The same authors found that 1,3-diCQA also significantly inhibits DPPH radicals more effectively than either trolox or caffeic acid. In addition to the abovementioned effects, 1,3-diCQA also demonstrated its ability to scavenge the oxidized by reactive oxygen species (ROS) (18). According to Danino et al. (23), 1,3-diCQA's antioxidant activity and ability to scavenge ROS make it a viable candidate for treating conditions, ranging from aging to degenerative disorders.

### Carotenoid Components

The different carotenoid components in six varieties of sweet potato leaves are presented in Table 4. Leaves of sweet potato cultivar “Blesbok” contained the highest levels of β-carotene (10.27 mg kg^−1^ DW) and zeaxanthin (5.02 mg kg^−1^ DW) compared to other domestic cultivars “Beauregard” and Peruvian cultivar “199062.1.” However, the Peruvian “199062.1” and “Beauregard” cultivars contained the lowest levels of β-carotene and zeaxanthin, respectively. In addition, lutein content was highest in leaves of cultivar “Bophelo,” followed by “Blesbok.” Conversely, the lutein content of Kenyan sweet potatoes varied from 285.7 to 446.6 g kg^−1^ on a dry weight basis was higher than the levels found in four South African cultivars, “Beauregard,” and Peruvian “199062.1.” Lutein cannot be synthesized in the body; it must be consumed from outside sources. Incorporating lutein into the diet of a consumer at an early age will reduce the severity of age-related macular degeneration (25). Incorporating Lutein into the diet of a consumer at an early age will reduce the severity of age-related macular degeneration. Identification of edible sources of lutein and fortification of foods with lutein could reduce the severity of age-related macular degeneration. Among dark vegetables, Menelaou et al. (26) found sweet potato leaves to be the highest source of lutein. Also, the results of the study showed that β-carotene content is determined by genotype. Despite this, indigenous plants, such as *Cleome hitra* (131.705 mg kg^−1^ DW), *Corchorus trilocularis* (54.43 mg kg^−1^ DW), *Moringa oleifera* (100-285 mg kg^−1^ DW), and *Solanum nigrum* (131.705 mgkg^−1^) (27) contain higher levels of β-carotene than sweet potato leaves investigated in this study. For men and women aged 19 to 50, the recommended dietary allowance (RDA) for vitamin A is 900 μg retinol activity equivalents (RAE) and 700 μg RAE, respectively (28). A 100 g of dried “Blesbok” sweet potato leaves contributes 9.51 and 12.23% of the RDA for Vitamin A for men and women, respectively. Alternatively, carotenoid levels in leafy vegetables depend on a number of factors, such as the cultivar, cultivar, the farming method, maturity, as well as environmental factors, such as light, temperature, and soil quality (8). Moreover, “Bophelo” was recommended for dual purpose use (use of both storage roots and leaves) due to the high potential contribution to β-carotene as well as iron and zinc.

**Table 4:** Comparison of different carotenoid components in the leaves of four South African sweet potato cultivars with the USA's “Beauregard” and Peru's “199062.1” cultivars on a dry weight basis.

	**Orange fleshed storage roots**			**Cream fleshed roots**		
Carotenoid components (mg/kg)	“Bophelo” leaves	“Beauregard” leaves	“199062.1” leaves	“Monate” leaves	“Ndou” leaves	“Blesbok” leaves
Lutein	9.50 ± 0.04a	2.62 ± 0.07c	1.29 ± 0.00e	1.29 ± 0.00e	1.88 ± 0.03d	7.19 ± 0.04b
Zeaxanthin	2.27 ± 0.06c	0.14 ± 0.01f	2.13 ± 0.04d	2.96 ± 0.06b	0.50 ± 0.01e	5.02 ± 0.02a
β-carotene	4.36 ± 0.10e	7.33 ± 0.52b	3.67 ± 0.09f	6.36 ± 0.06c	6.21 ± 0.20d	10.27 ± 0.20a
% Vit A RDA male >14/ 100 g	4.04	6.79	3.40	5.89	5.75	9.51
% Vit A RDA female >14 per100 g portion	5.19	8.73	4.37	7.57	7.39	12.23

*Means followed by the same letter within the row are not significantly different at p < 0.05*.

## Conclusion

Using a practical metabolomic chemometrics tool, we discriminated between leaves from four sweet potato cultivars grown in South Africa, USA cultivar “Beauregard,” and Peruvian cultivar “199062.1” using their phenolic compounds. It is evident from this study that phenolic compounds are almost identical between the leaves of three diverse groups of sweet potato cultivars, but they differ in composition. The use of sweet potato leaves as leafy vegetables is a new concept in most countries, but, because of its functional properties, it is destined to become a niche market. Additionally, it would be ideal to recommend suitable local sweet potato cultivars that can be used as leafy vegetables in Sub-Saharan Africa. Therefore, breeding and commercialization of sweet potato cultivars “Bophelo” and “Blesbok” for leafy vegetable consumption are encouraged due to their high caffeoylquinic acid and carotenoids content, respectively. It is important to investigate the palatability and antinutritive components of the leaves.

## Data Availability Statement

The original contributions presented in the study are included in the article/Supplementary Material, further inquiries can be directed to the corresponding author/s.

## Author Contributions

CP performed the experiment, generated the data, and wrote some parts of this manuscript. SL developed the sweet potato cultivars, provided the leaves, advised on the investigations, and contributed to the write-up of the article. TS was responsible for HPLC analysis for carotenoid, visualized and validated the data for phenolic compounds, and interpreted the chromatogram. VM performed the metabolomic chemometric analysis and validated the phenolic compounds. DS conceptualized the research, supervised the CP, and improved the article further. All authors contributed to the article and approved the submitted version.

## Funding

This work is based on research supported in full by the National Research Foundation of South Africa (Grant No. 98352) for Phytochemical Food Network to Improve Nutritional Quality for Consumers.

## Conflict of Interest

The authors declare that the research was conducted in the absence of any commercial or financial relationships that could be construed as a potential conflict of interest.

## Publisher's Note

All claims expressed in this article are solely those of the authors and do not necessarily represent those of their affiliated organizations, or those of the publisher, the editors and the reviewers. Any product that may be evaluated in this article, or claim that may be made by its manufacturer, is not guaranteed or endorsed by the publisher.
