# Supplementary material for: An Evaluation of Phenolic Compounds, Carotenoids, and Antioxidant Properties in Leaves of South African Cultivars, Peruvian 199062.1 and USA's Beauregard
Source: Front Nutr. 2021 Nov 26;8:773550. doi: 10.3389/fnut.2021.773550 (PMC8662696; doi:10.3389/fnut.2021.773550)
Supplement: Supplementary file 1 [file Data_Sheet_1.pdf]

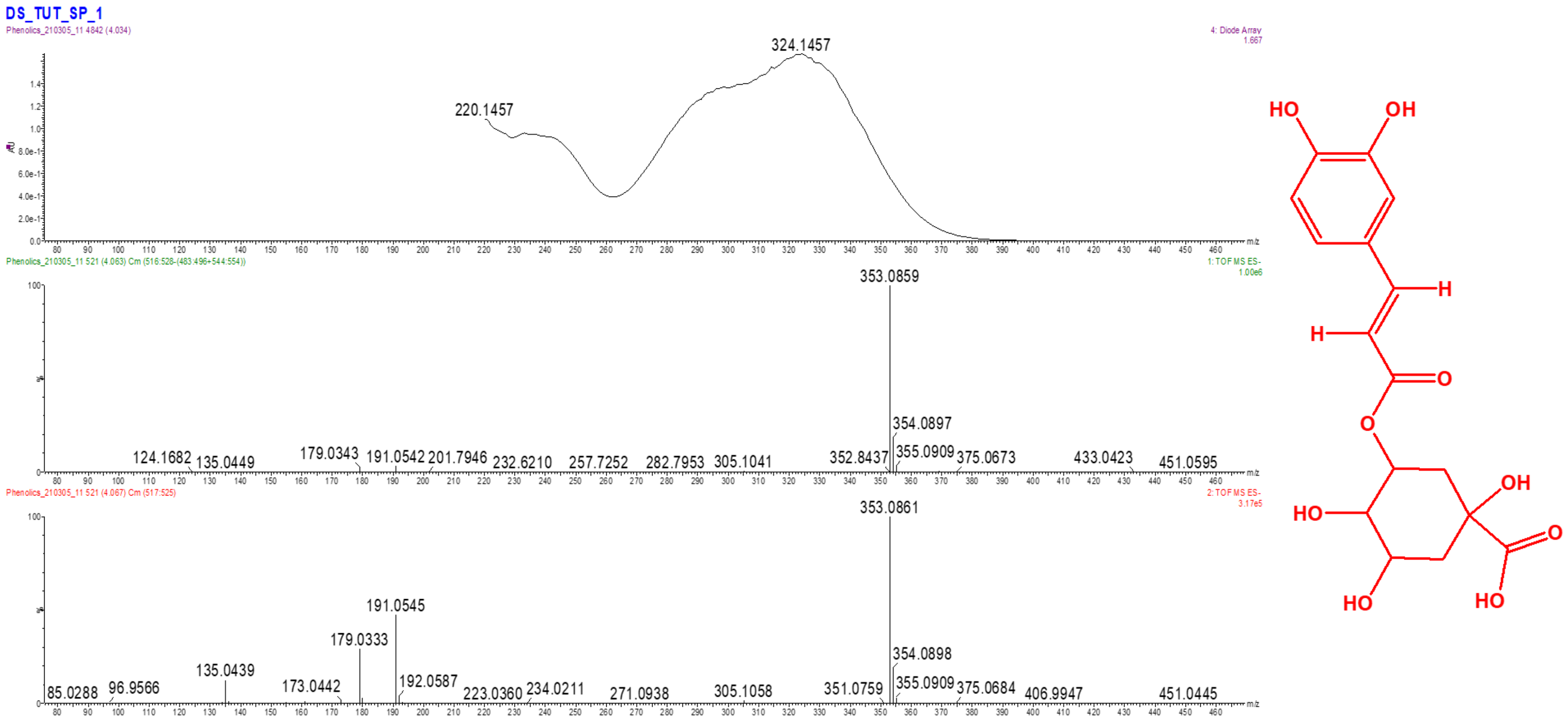

Supplementary (S) Figure 1A: UV absorption spectrum of neochlorogenic acid overlaid on the MS and MS/MS spectrum adjacent to the chemical structure (in red)

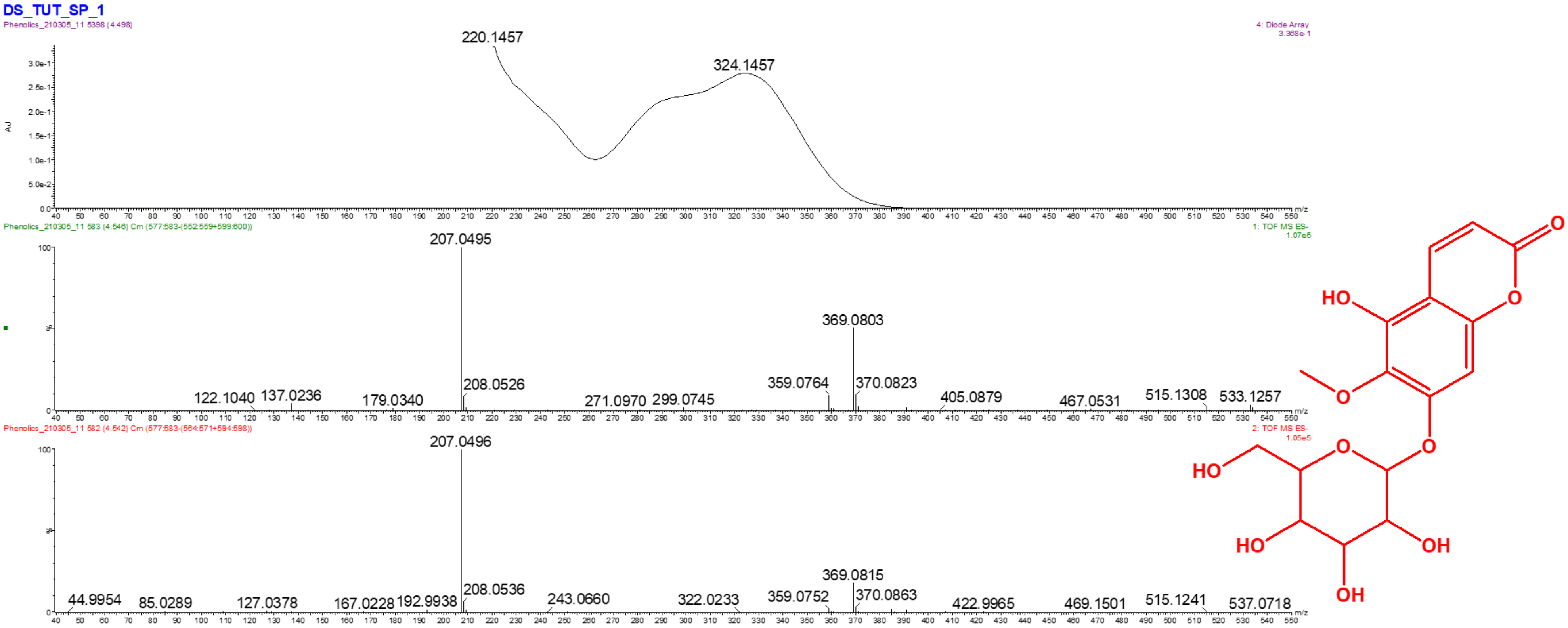

S Figure 1B: UV absorption spectrum **of 5 hydroxy-6 methoxycoumarin 7 glucoside overlaid on its MS and MS/MS spectrum adjacen to its chemical structure(in red)**

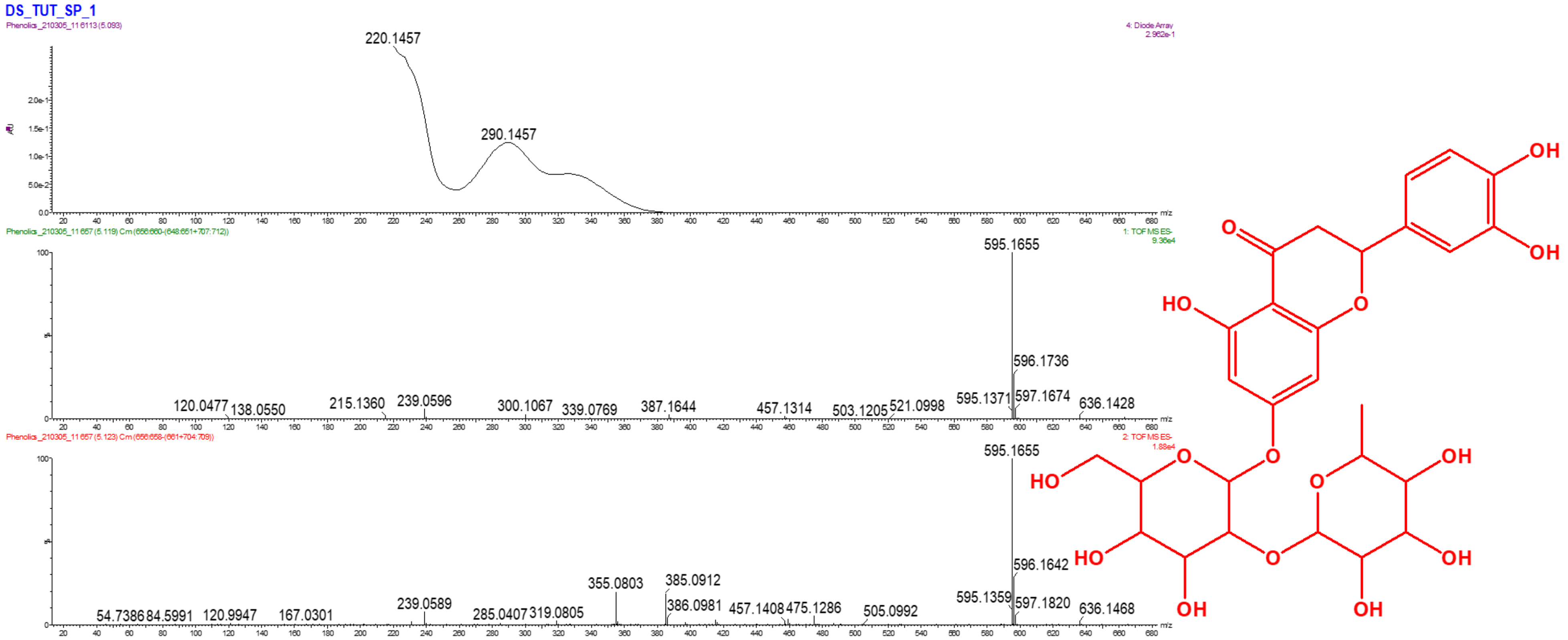

S Figure 1C: UV absorption spectrum of Eriodictyol 7-O-neohesperidoside overlaid on its MS and MS/MS spectrum adjacent to its chemical structure (in red).

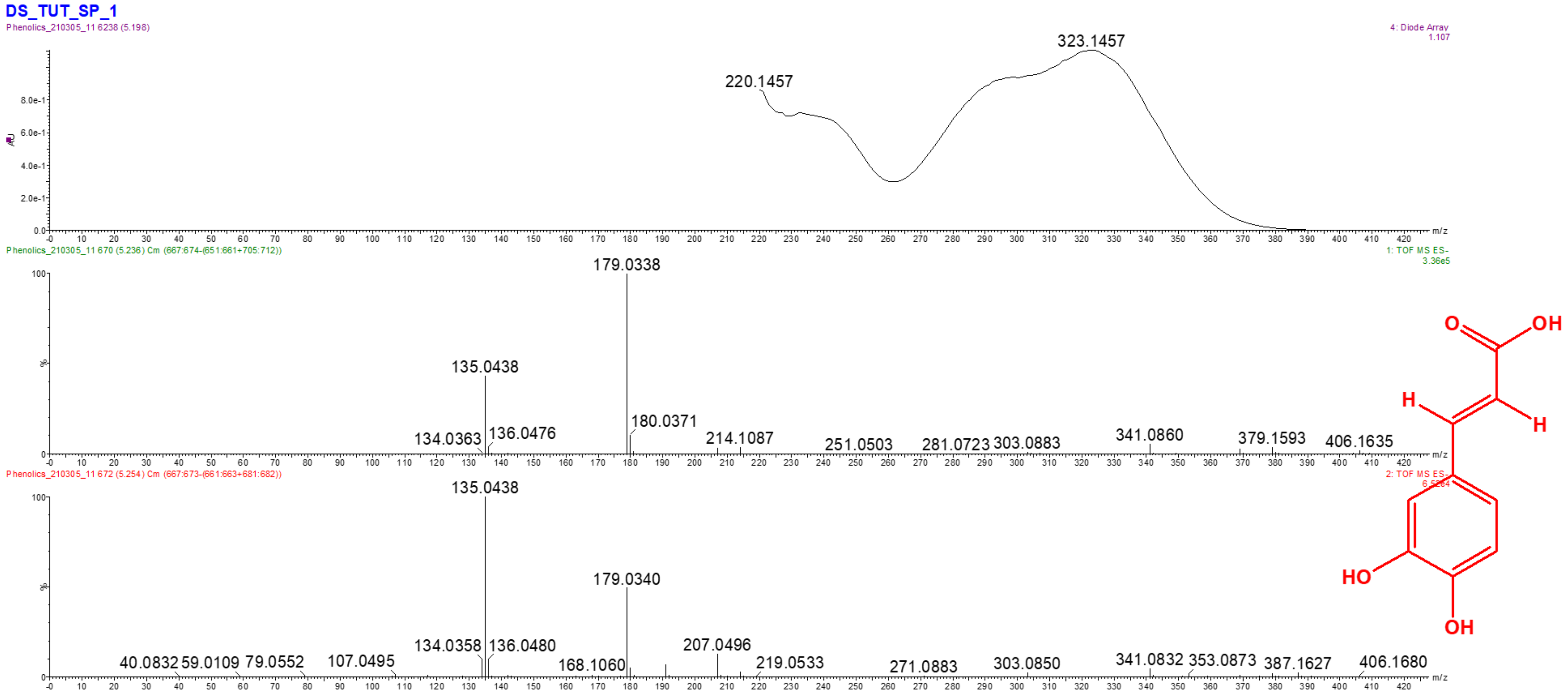

S Figure 1D: UV absorption spectrum of caffeic acid overlaid on its MS and MS/MS spectrum adjacen to its chemical structure (in red)

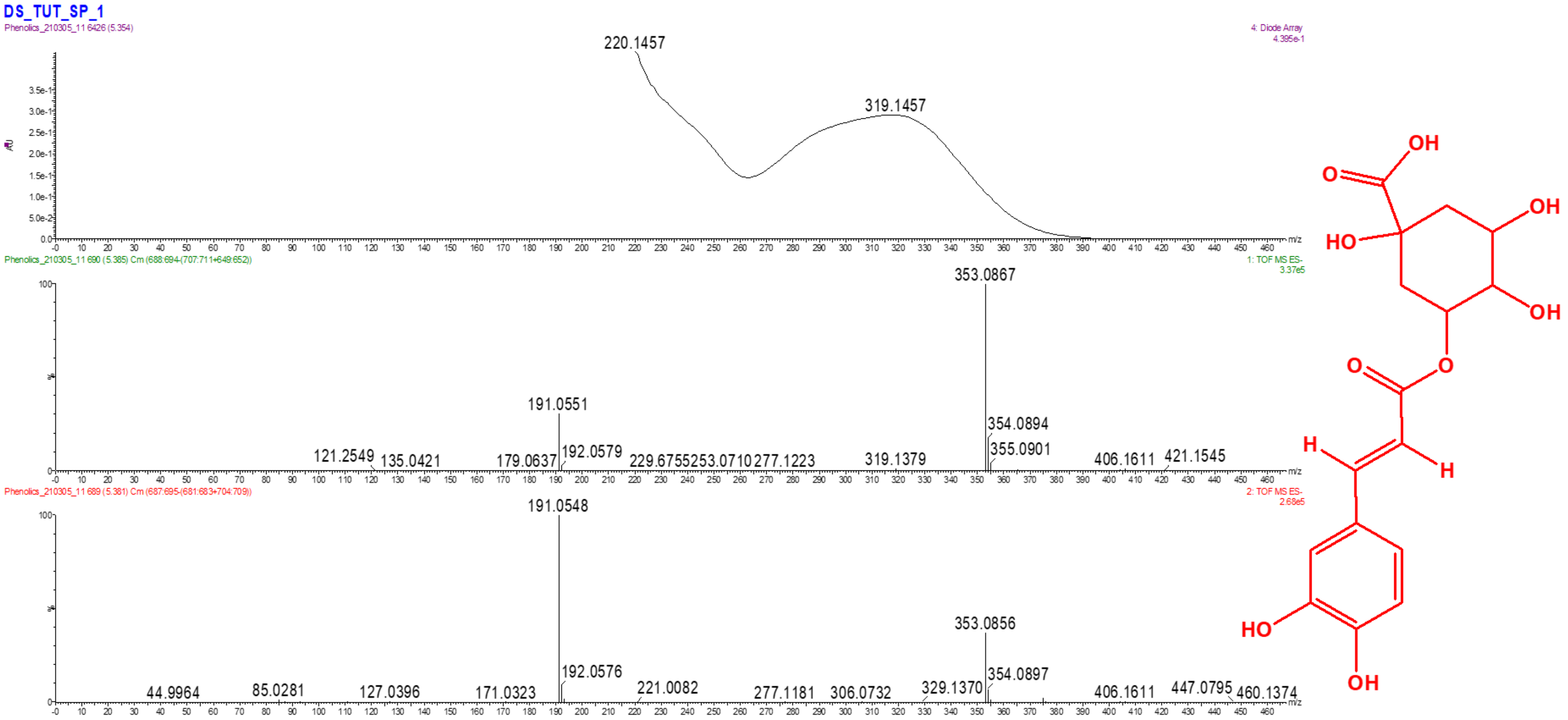

S Figure 1E: UV absorption spectrum of chlorogenic acid overlaid on its MS and MS/MS spectrum adjacent to its chemical structure (in red)

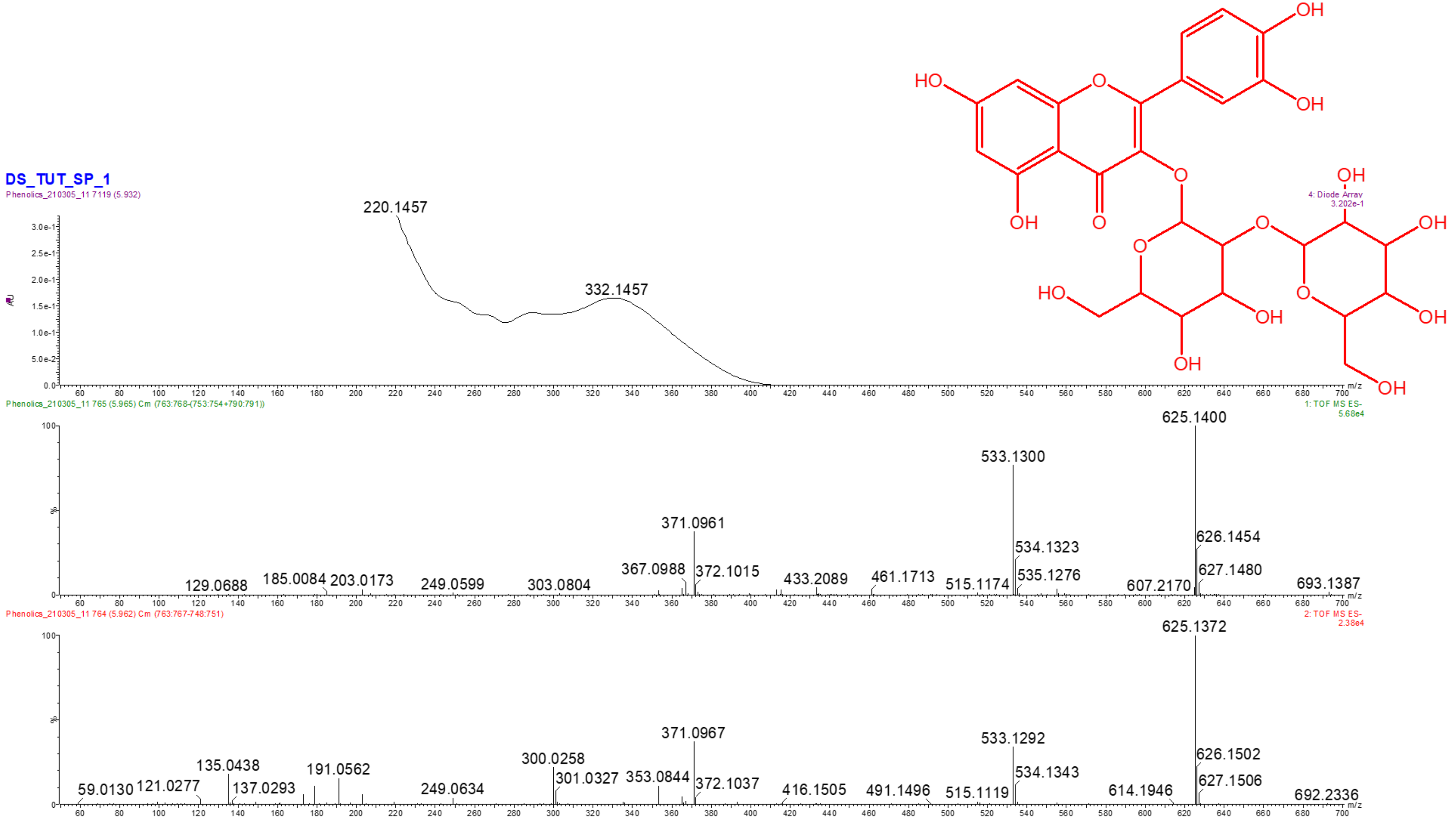

S Figure 1F: UV spectrum of quercetin 3-glucosyl-(1->2)-galactoside overlaid on its MS and MS/MS and its chemical structure is shown (in red)

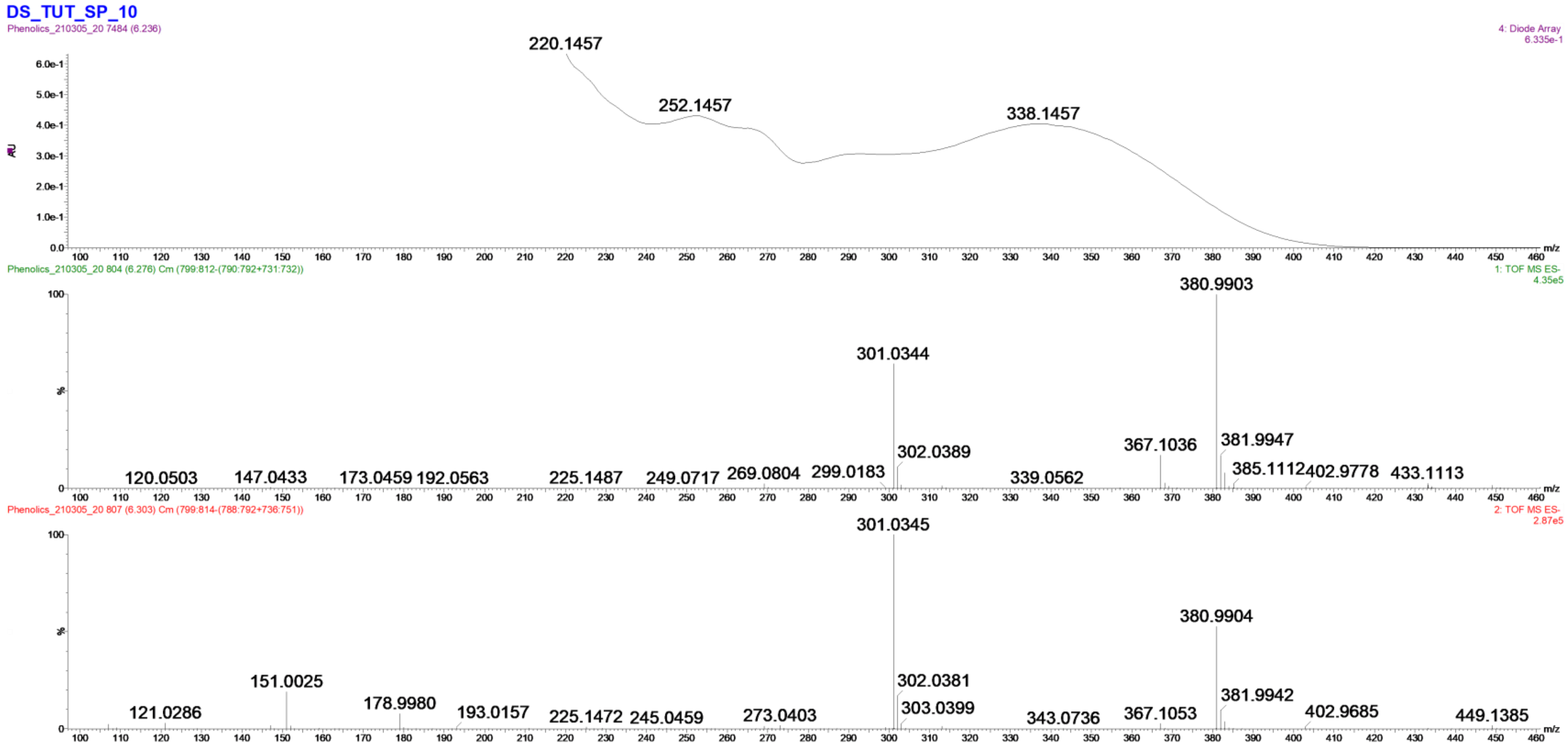

S Figure 1G: UV spectrum of a quercetin derivative overlaid on its MS and MS/MS spectrum

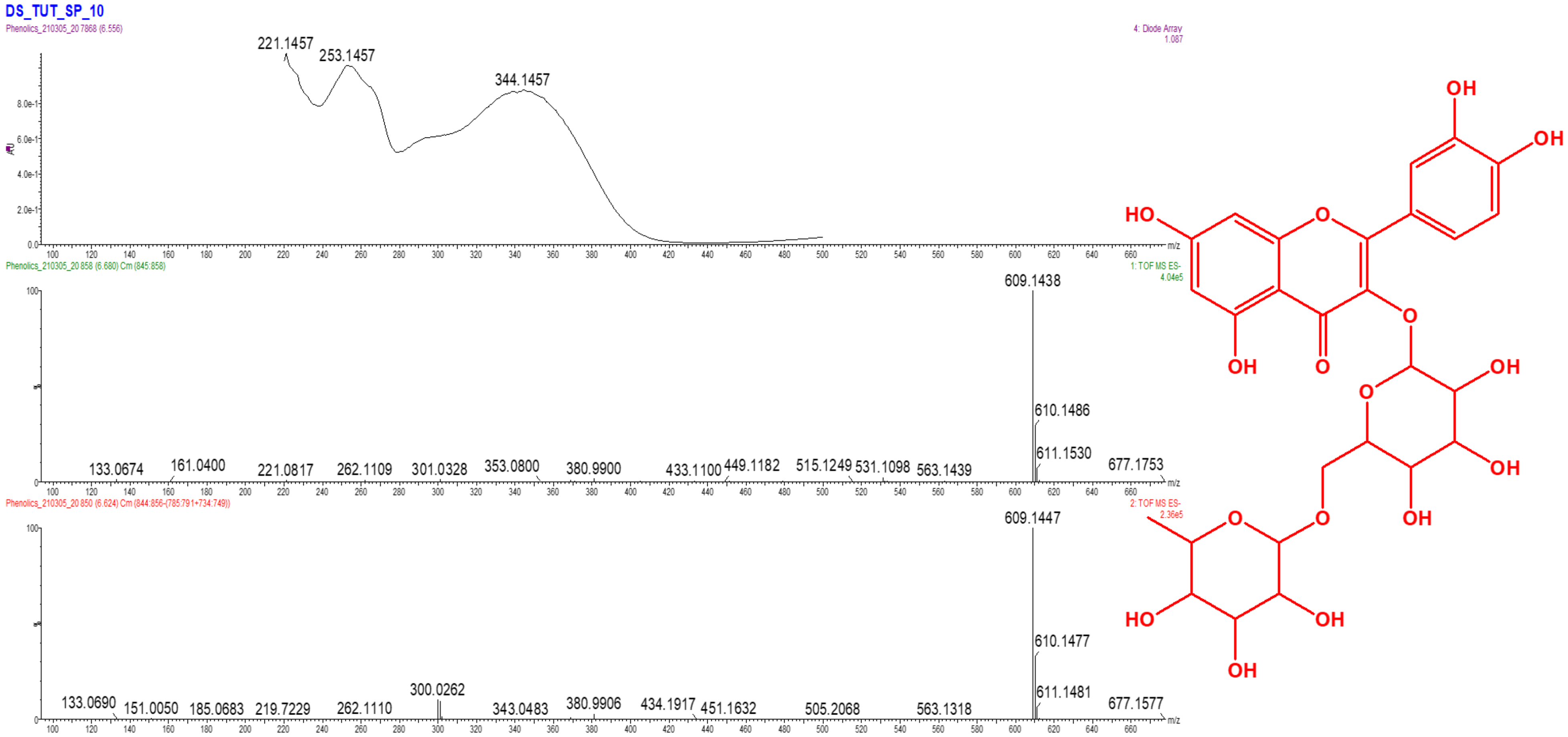

S Figure 1H: UV absorption spectrum of rutin overlaid on its MS and MS/MS spectrum adjacent to its chemical structure

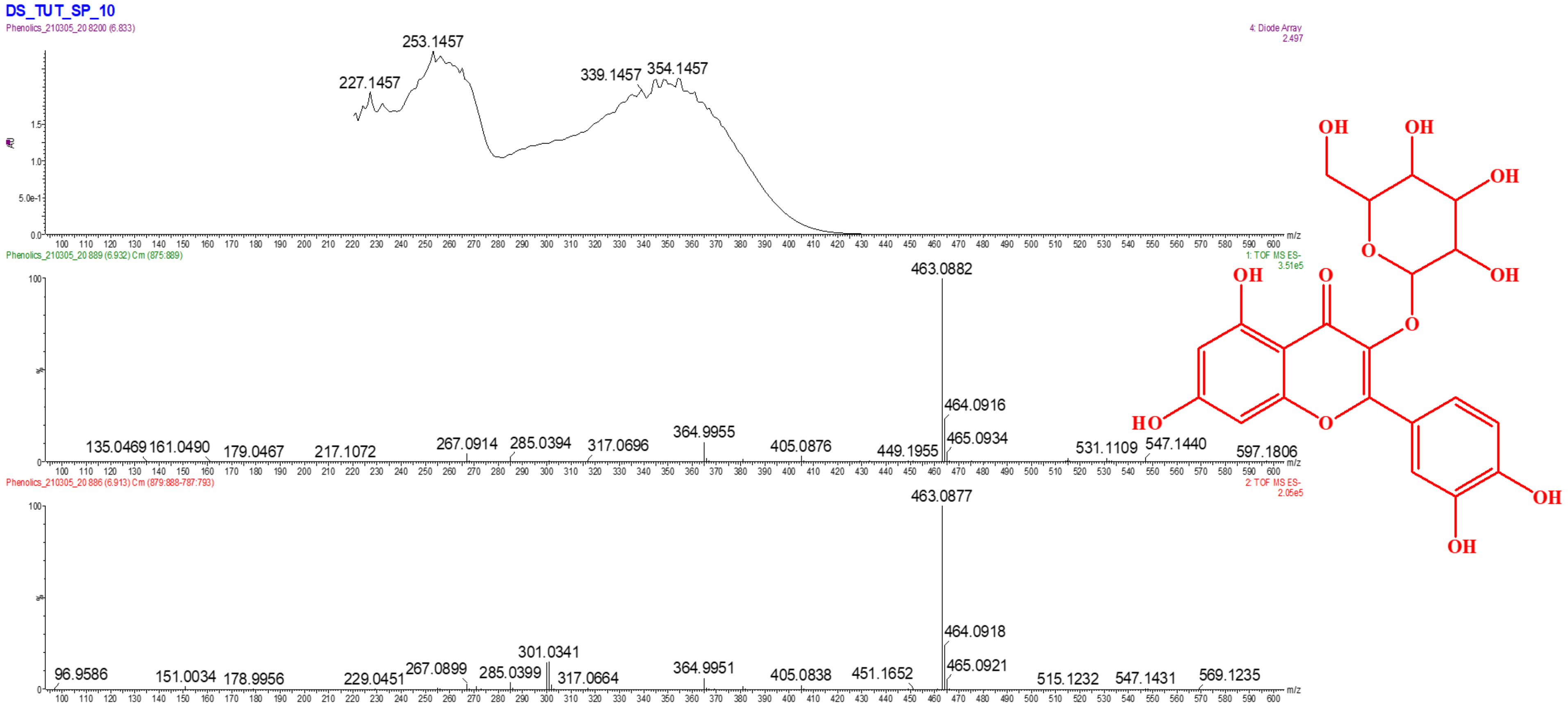

S Figure 1J: UV absorption spectrum of quercetin 3 galactoside overlaid on its MS and MS/MS spectrum adjacent to its chemical structure

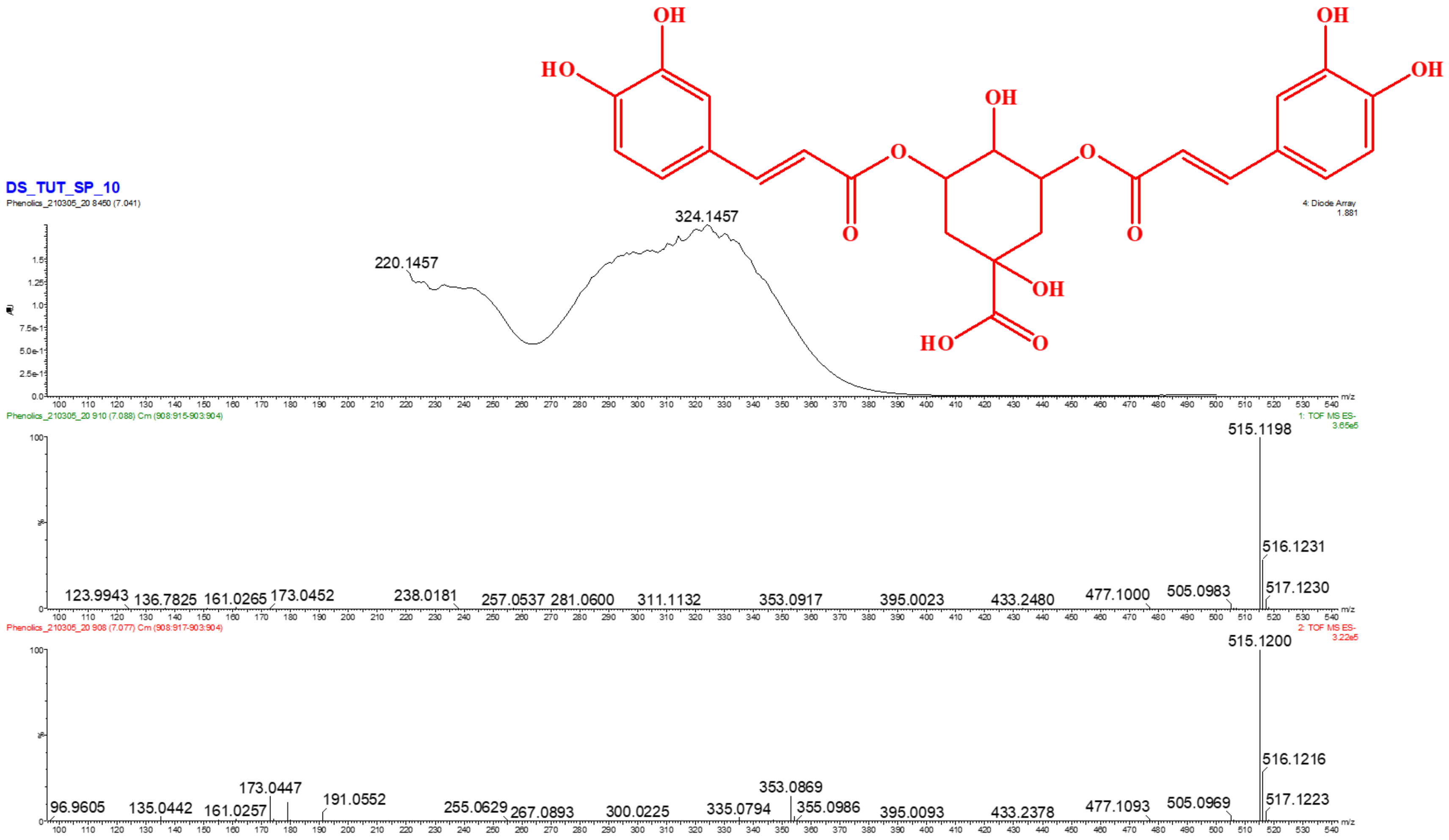

S Figure 1K: UV absorption spectrum of 3,5 dicaffeoylquinic acid overlaid on its MS and MS/MS spectra and its chemical structure is shown (in red)

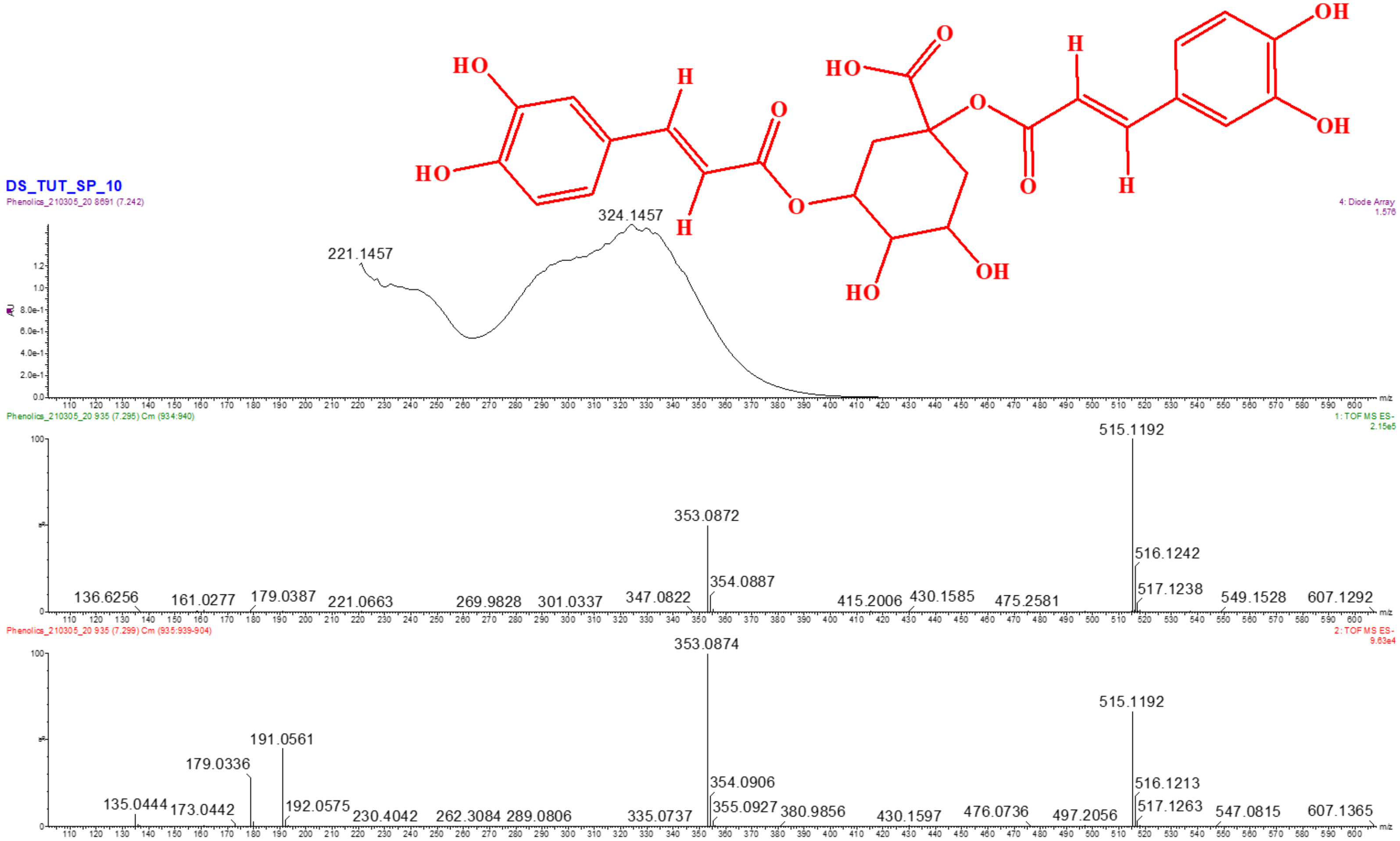

S Figure 1L: UV spectrum of 1,3 dicaffeoylquinic acid overlaid on its MS/MS spectrum

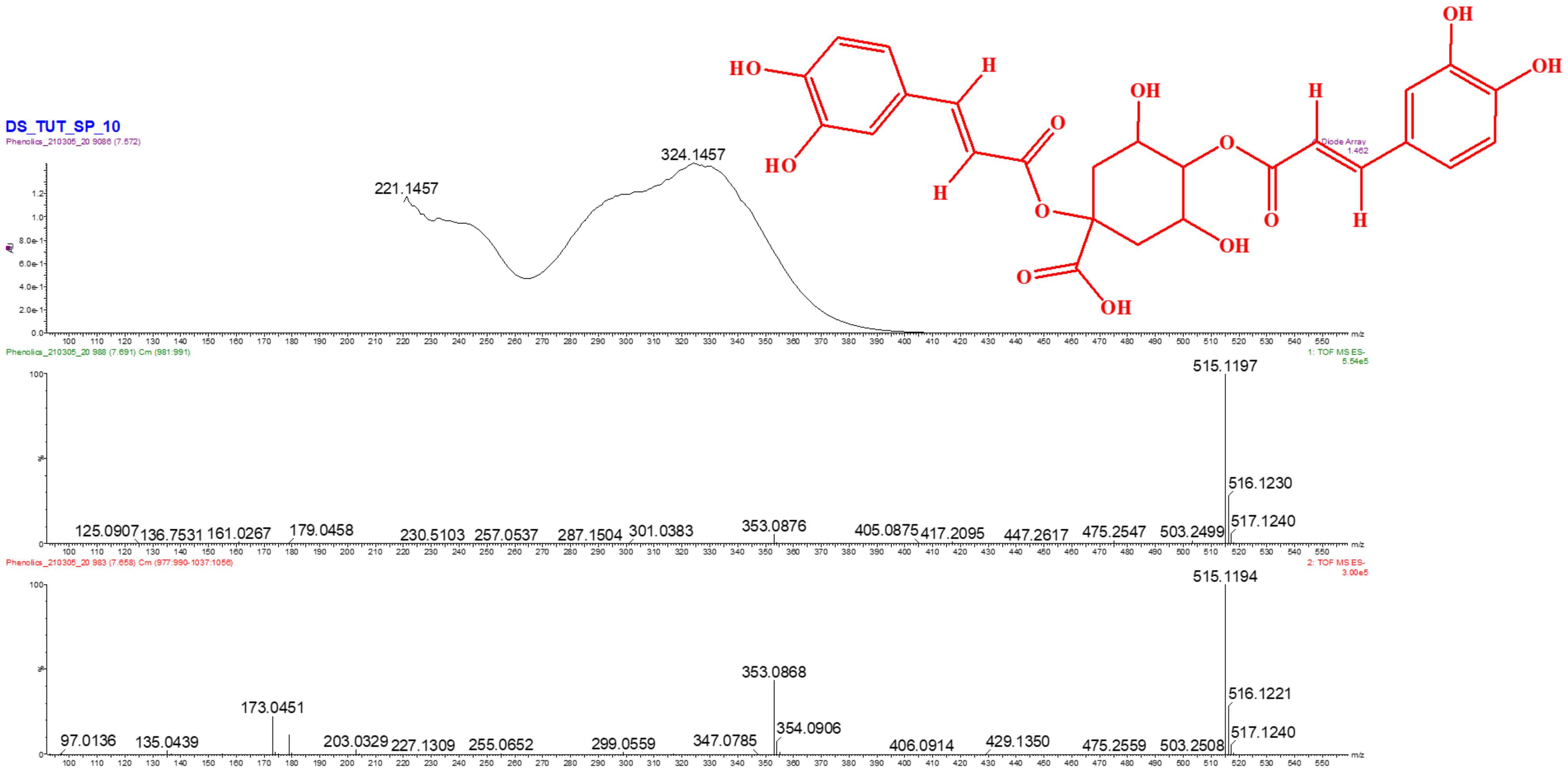

S Figure 2 : UV spectrum of 1,4 dicaffeoylquinic acid overlaid on its MS and MS/.MS spectrum and its chemical structure (in red)

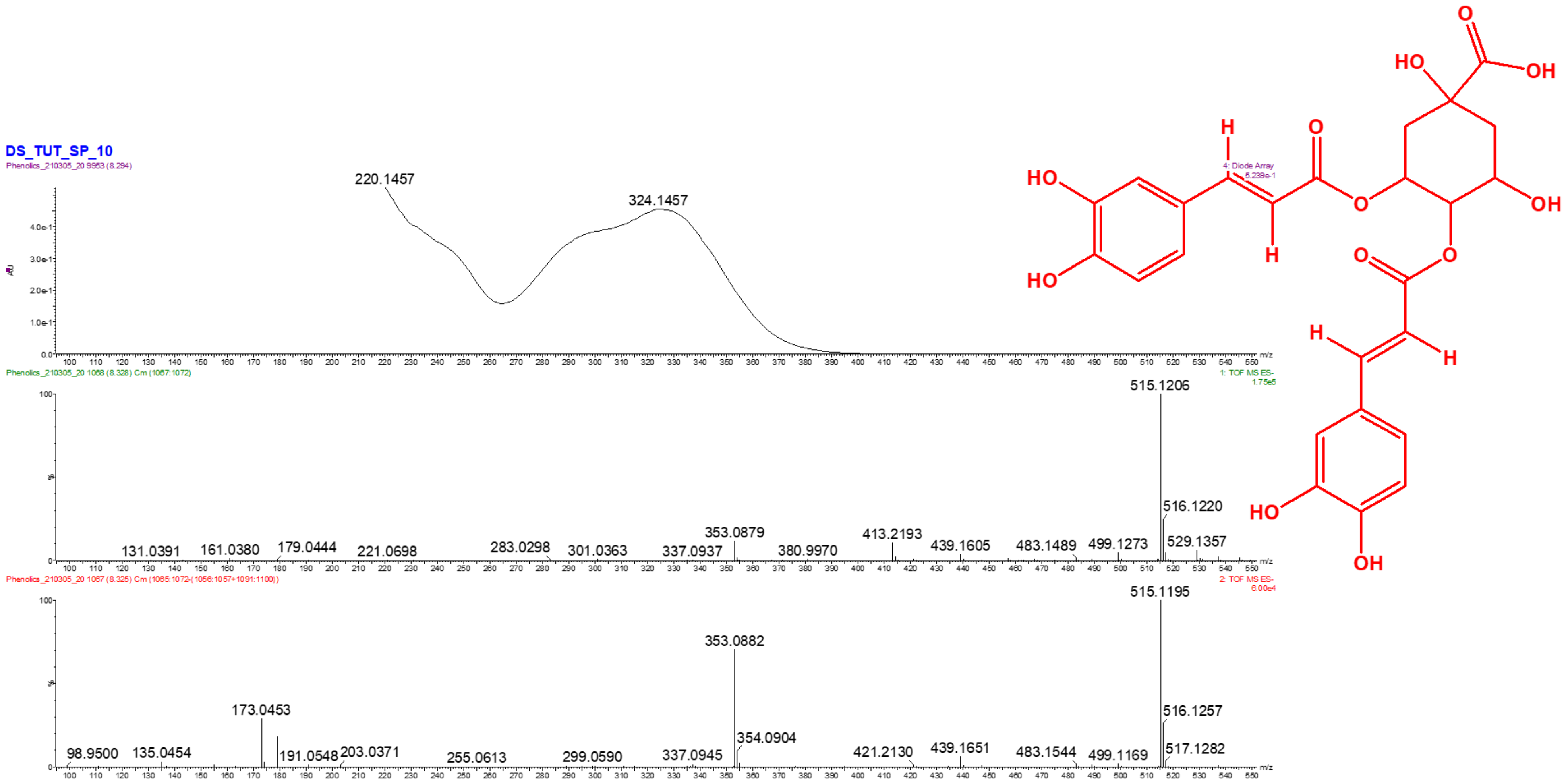

S Figure 1N: UV spectrum of 4,5 dicaffeoylquinic acid overlaid on its MS and MS/MS spectrum adjacent to its chemical structure (in red)
